# Reciprocal associations between housing instability and youth criminal legal involvement: a scoping review

**DOI:** 10.1186/s40352-022-00177-7

**Published:** 2022-04-08

**Authors:** Lars Almquist, Sarah Cusworth Walker

**Affiliations:** 1 Department of Health Systems and Population Health, University of Washington, Seattle, United States; 2 Department of Psychiatry and Behavioral Sciences, University of Washington, Seattle, United States

**Keywords:** Juvenile justice, Social determinants, Housing, Homelessness, Youth, Review

## Abstract

**Background:**

Youth experiencing homelessness have disproportionate contact with the criminal legal system. This system contact represents a critical inflection point for enhancing risk or opportunities for stabilization; however, the policy and scholarly traditions examining the criminal legal system have not traditionally incorporated housing or other social determinants as a central focus of intervention.

**Methods:**

We conducted a scoping review using PRISMA-ScR guidelines to examine how the research literature is currently addressing housing within the context of youth involvement in the legal system. Databases searched included PubMed, Web of Science, and Academic Search Complete. Google Scholar was used to identify papers not indexed in the academic databases of interest. Database searches were conducted between September and December 2019 and articles were restricted to those published in English between the year 2000 and 2019. Key study components extracted included demographic information regarding each sample, type of article, study methodology, direction of effects of interest, outcome measures and primary findings, as well as theoretical frameworks engaged by the authors.

**Results:**

The search results returned 2154 titles for review. After screening all 2154 titles, 75 met eligibility for inclusion. Abstract reviews were conducted for all 75 papers. 36 abstracts met eligibility criteria and underwent full-text review. Ultimately, 29 articles satisfied eligibility criteria and were included in this scoping review.

**Conclusions:**

Publications are primarily focused on the social epidemiology of risk factors and behaviors determining youth justice contact, but relatively less so on studies of interventions targeting youth delinquency, crime reduction, or recidivism that included housing support. The lack of continuity in theorizing from epidemiology to applied science in this area represents a gap in the literature that is likely reducing the effectiveness of interventions to interrupt patterns of legal system contact for youth. Integrating a public health framework that emphasizes the upstream social determinants leading to contact with the youth justice system would represent a paradigm shift for the field that would have beneficial effects on long term health outcomes for youth.

## Background

Homelessness exerts a devastating effect on the physical, behavioral, and psychosocial health of youth (Bender et al., 2014; Hodgson et al., 2013; Kaufman & Widom, 1999; Medlow et al., 2014). The number of adolescents ages 13–17 experiencing homelessness or runaway in the United States exceeds 660,000 annually (Morton et al., 2018), with projected increases over the coming years (Wiltz, 2017). One of the correlates of youth housing instability is increased contact with the youth criminal legal (hereafter juvenile justice) system. A disproportionate number of youth experiencing homelessness will have contact with law enforcement compared to stably housed youth (Baron, 2016; Chapple et al., 2004; Chen et al., 2006; Edalati & Nicholls, 2019; Ivanich & Warner, 2019; McCandless, 2018; Omura et al., 2014; Snyder et al., 2016; Thrane et al., 2008; Walker et al., 2018; Yoder et al., 2014). This system contact represents a critical inflection point that presents varied risks and opportunities for stabilization (Nordess et al., 2002; Rodriguez, 2007; Walker & Herting, 2020). These impacts are largely unstudied, as the policy and scholarly traditions examining the juvenile justice system have not traditionally incorporated housing or other social determinants as a central focus of practical theorizing. Findings from the literature on adult exposure to incarceration and homelessness reveal a revolving door of system involvement and housing insecurity. Incarcerated individuals are often released into situations of homelessness, which is then associated with recidivism and readmission to the criminal legal system (Lutze, Rosky & Hamilton, 2014). Integrating a public health framework within youth legal and justice system work would represent a paradigm shift for the field that could have beneficial effects on long term health outcomes for youth.

### Social determinants and cumulative health risk

Within the field of public health, a lens of “social determinants” of health (Braveman et al., 2011; Braveman & Gottlieb, 2014; Marmot et al., 2008; Terris, 1968) is used to frame the comprehensive ecology of risk and protective factors that drive health behaviors and access to resources that contribute, either passively or directly, to various health outcomes. Within this framework, individual exposures and outcomes do not occur in isolation, but rather occur within the wider context of the entire course of an individual’s life and the social and environmental contexts in which that life is lived (Ben-Shlomo & Kuh, 2002; Currie et al., 2012; Link & Phelan, 1995). Individuals may be disproportionately exposed to certain phenomena or to experience particular outcomes depending upon the developmental period within the life course. These time frames are referred to as “sensitive periods” or “critical periods” (Keyes & Galea, 2016; Kuh et al., 2003; Wood et al., 2018). Adolescence, encompassing puberty and the developmental transition into adulthood, is one such period where risks of various kinds are heightened compared to the rest of the life course (Blakemore & Mills, 2014; Moffitt, 2003; Viner et al., 2012; Viner et al., 2015). This period is marked by compounding personal, social, biological, emotional, and hormonal changes experienced simultaneously, forcing the youth to navigate an acutely complex period in their life without the benefit of fully developed mental, emotional, financial, vocational, or relational supports typically obtained later in life.

Heightened risk in one area creates higher risk in other health areas. In public health scholarship, these phenomena are referred to as chains of risk (Ben-Shlomo & Kuh, 2002). Risks accumulate over the life course, compounding physical and psychological effects (Keyes & Galea, 2016). Individuals experiencing accumulating risks, particularly within the sensitive period of adolescence, are disproportionately more likely to have contact with the justice system as well as other unfavorable health outcomes (Walker et al., 2018).

### Intervention frameworks and delinquent youth

Traditionally, intervention frameworks for justice-involved youth primarily focus on cognitive and emotional regulation skills (Cleare, 2000), family conflict (Henggeler & Schoenwald, 2011), and positive youth development (Durlak et al., 2007), rather than social determinants of a youth’s justice involvement, such as their housing status. This focus persists despite dominant criminological frameworks suggesting that crime may arise from a combination of social strain (e.g., abusiveness, trauma, housing) and the ability to cope with the strain legally (Thaxton & Agnew, 2018). Interventions have almost exclusively focused on the cognitive coping aspects of this explanatory model rather than seeking to eliminate or reduce social strain directly. Even within ecological models, housing tends to be marginalized as an explanatory factor. For example, a systematic review of re-offending risks conducted by Jacobs et al. (2020) does not include housing status beyond single vs dual parent homes and income level. This is consistent with other research examining risk and protective factors in legal system-involved youth, in which housing status is rarely taken into account as a primary factor related to either risk or responsivity (Olver et al. 2009; Vincent et al., 2012).

Increasingly, public health frameworks recognize that social determinants are critical predictors of long-term health. To date, there has been little inquiry into how legal-system involvement and subsequent youth interventions should similarly account for these factors. We conducted a scoping review to examine how the research literature is currently addressing housing in the context of youth legal involvement. A scoping review is a method of critically observing how a scholarly topic is being studied and was appropriate given our interest in better understanding the extant literature.

### Purpose of this review

Consistent with the purpose of a scoping review (Pham et al., 2014; Tricco et al., 2018), the current study aimed to evaluate the state of the literature regarding an integration of a housing lens within the juvenile delinquency research literature. The review emerged from three primary research questions: 1) what is the state of the literature regarding the impact of youth housing instability and justice system involvement as determinants of one another?; 2) what are the theoretical frameworks informing this research?; and 3) what implications do these findings hold for the next generation of youth justice-focused interventions? To address these questions, we first present the methodology guiding our search of the existing literature. We then articulate the characteristics of studies included in the review and assess the theoretical frameworks informing existing research regarding the intersection of housing instability and juvenile justice contact. Finally, we discuss implications for system-level interventions and future research opportunities.

## Methods

### Identification of the literature

Scoping reviews are a methodologically rigorous approach to describing the published literature on a topic of interest (Arksey & O'Malley, 2005). We used the most recent guidance for conducting high quality scoping reviews, drawing from foundational literature (Arksey & O'Malley, 2005; Peters et al., 2020) and updated methods (Levac et al., 2010; Pham et al., 2014)). This involved a multi-step, iterative process to identify the framing questions, refine search terms, confirm the scope of the review, and establish selection criteria to meet the goals of the review.

### Search strategy

**Fig. 1:**
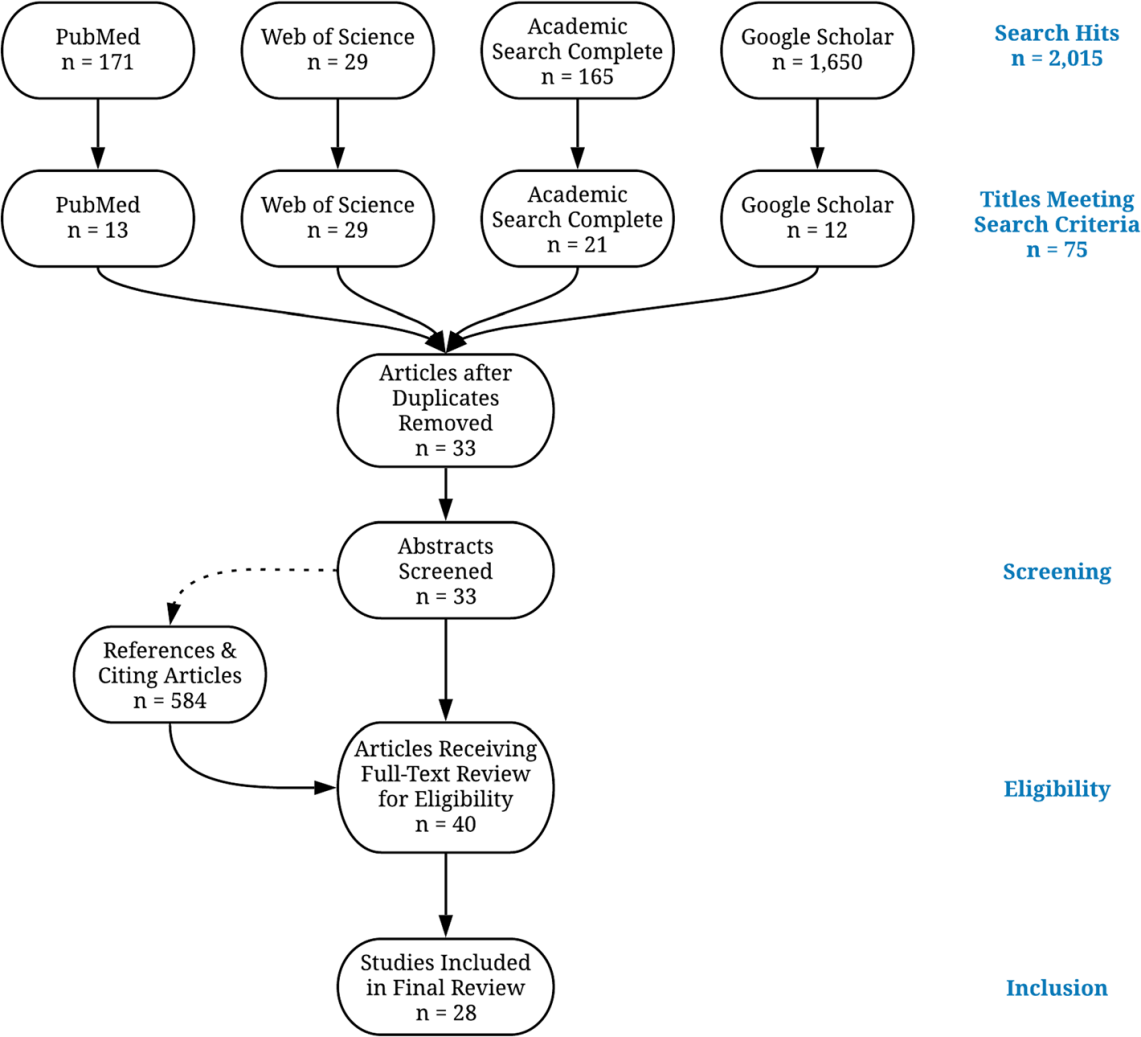
Article Screening and Inclusion Eligibility

The search strategy was motivated by an interest in understanding how the social determinants of health literature influences the epidemiological and intervention juvenile justice literature. The two authors met multiple times to discuss the appropriate search terms to identify the extant literature in this area and the first author conducted the searches. Final search terms included combinations of the following terms: *housing, housing stability, homelessness, juvenile court, juvenile justice, juvenile detention, criminal justice, youth, and social determinants*. Specific combinations of search terms may be found in Table 1. A secondary search was conducted using the subject term *homeless* in combination with the above terms. Databases used in the search included PubMed, Web of Science, and Academic Search Complete. Google Scholar was also searched to identify papers that were not indexed in the academic databases of interest. Database searches were conducted between September and December 2019 and articles were restricted to those published in English between the year 2000 and 2019. Additional articles citing the identified publications were reviewed in January and February 2020. Final search terms were developed through an iterative process designed to refine searches to capture the explicit engagement of housing instability and involvement with the juvenile legal system. Article selection and synthesis were conducted based on the Preferred Reporting Items for Systematic reviews and Meta-Analyses extension for Scoping Reviews (PRISMA-ScR) checklist. A PRISMA-ScR Flow Diagram is found in Fig. 1.

**Table 1:** Search Terms

housing stability AND juvenile court	
housing stability AND juvenile justice	
housing stability AND juvenile detention	
housing AND juvenile justice	
housing AND juvenile court	
housing AND juvenile detention	
social determinants AND juvenile justice	
social determinants AND juvenile detention	
social determinants AND juvenile court	
criminal justice AND youth AND housing	
criminal justice AND youth AND homelessness	

*Each combination of terms was entered into PubMed, Web of Science, Academic Search Complete, and Google Scholar and combined with the search term “homeless”*

### Selection criteria

Articles were included if they addressed youth housing instability and involvement with the juvenile justice system. Youth were defined as individuals ages 18 and younger. Publications including participants over age 18 were included for review if the study addressed the housing instability or justice involvement of participants when they were minors. Given the specific focus on youth housing instability and justice involvement as determinants of one another, papers focused on youth exclusively within institutional systems, such as foster care or those housed within detention centers, were excluded. Studies focused on justice involvement for youth transitioning out of foster care or housing instability for youth exiting the justice system were included for review. Non-empirical papers with a narrow focus on policy analysis, proposals, or critiques were used to identify additional eligible papers.

Combinations of search terms were entered into three publication databases (PubMed, Academic Search Complete, and Web of Science). Titles identified via all databases were screened for relevance based on inclusion criteria established by the authors. Additionally, queries were made in Google Scholar to capture publications that were not indexed in the three databases. Given the extensive results produced by Google Scholar, title review was limited to the first 150 publications returned by each search query. Abstracts were reviewed for all unduplicated titles meeting inclusion criteria. Publications with abstracts meeting selection criteria underwent a full-text review, with relevant publications ultimately selected for coding and inclusion in this review. The results were managed in an Excel database with links to access full text articles.

### Data extraction

Data were extracted from each review based upon a protocol created by the authors. Extracted data included:

- Review identifiers: full citation, including author, year of publication, article title, and place of publication
- Setting and population: geographical location of primary author, geographical location of target population, age range, female proportion of target population
- Methodology: study design, type of article (epidemiological/risk assessment, systematic review, policy argument, ethnography, observational study, program evaluation, etc.), theories influencing research
- Direction of the effect of interest (housing instability as a determinant of justice contact, justice contact as a determinant of housing instability, or co-occurring phenomena)
- Outcome measures and primary findings

The first author completed a first round of data extraction from articles included in the review. Both authors reviewed extracted data and identified areas where data needed to be re-extracted for clarity or to better align with the coding scheme.

## Results

The search results returned 2154 titles for review. Given the extensive results returned by searches in Google Scholar, title reviews were limited to the first 150 items returned by each independent query (*n* = 1650). After screening all 2154 titles, 75 met eligibility for inclusion. Abstract reviews were conducted for all 75 papers. Of these publications, 36 abstracts met eligibility criteria. A review of the titles of the 584 publications citing these 36 papers yielded seven additional studies for consideration. Full-text reviews were conducted for the resulting 40 papers meeting eligibility criteria. After full-text review, 29 articles satisfied eligibility criteria and were included in this scoping review. A breakdown of these articles may be found in Table 2.

**Table 2:** Overview of Included Studies

Author	Study Design	Type of Paper	Sample Size (% Female)	Target Population	Population Location	Age Range (Years)	Direction of Effect	Theories Identified
Baron (2008) [1]	Quantitative (Cross-Sectional)	Risk/Epi	400 (33.8%)	Homeless street youth	Ontario, Canada	13–24	H → J	General Strain Theory
Baron (2016) [2]	Quantitative (Cross-Sectional)	Risk/Epi	400 (36%)	Homeless street youth	Large western city, Canada	16–24	H → J	Self-Control Theory
Boyd et al. (2016) [3]	Ethnography	Ethnography	75 (44%)	Street-involved, substance using youth	Vancouver, Canada	14–26	H → J	
Britton & Pilnik (2018) [4]	Argument	Argument	N/A	System-involved youth	N/A	N/A	J → H	
Chapple et al. (2004) [5]	Quantitative (Cross-Sectional)	Risk/Epi	602 (60%)	Homeless and runaway youth	Iowa, Kansas, Missouri, Nebraska	12–22	H → J	
Chen et al. (2006) [6]	Quantitative (Cross-Sectional)	Risk/Epi	428 (56.3%)	Homeless and runaway adolescents with mental health disorders	Iowa, Kansas, Missouri, Nebraska	16–19	H → J	
Courtney et al. (2019) [7]	Quantitative (Program Eval.)	Interventions	1322 (48.0%)	System-involved (custodial care or juvenile justice) youth transitioning to adulthood	Tennessee	18–24	H → J	
Crawford et al. (2018) [8]	Quantitative (Cross-Sectional)	Risk/Epi	1420 (52%)	Youth transitioning from foster care to adulthood	Southwestern U.S. state	16–17	H → J	
Edalati & Nicholls (2019) [9]	Systematic Review	Systematic Review	13,123 (51.7%)	Homeless individuals with childhood abuse and neglect	Varies by publication	12–66	H → J	
Ivanich & Warner (2019) [10]	Quantitative (Longitudinal)	Risk/Epi	428 (60.2%)	Homeless youth	Iowa, Kansas, Missouri, Nebraska	16–19	H → J	Focal Concerns Theory
Jackson et al. (2017) [11]	Quantitative (Longitudinal)	Risk/Epi	1280 (48%)	Children with adverse housing conditions	20 U.S. cities	8–11	H → J	General Strain Theory
Jeanis et al. (2019) [12]	Quantitative (Cross-Sectional)	Risk/Epi	29,204 (29.2%)	Delinquent runaway youth	Florida	12–18	H → J	
Kolivoski et al. (2017) [13]	Quantitative (Longitudinal)	Risk/Epi	794 (50.8%)	Child-welfare-involved youth	Large county in Mid-Atlantic state	12–22	H → J	
Kort-Butler & Tyler (2012) [14]	Quantitative (Cross-Sectional)	Observational	249 (55.0%)	Homeless and runaway youth	3 Midwestern cities	14–21	Co-Occurring	
McCandless (2018) [15]	Ethnography	Ethnography	18 (% unknown)	LGBT youth (interviewed as adults)	6 locales across the U.S. (unspecified)	18+	H → J	
Narendorf et al. (2020) [16]	Quantitative (Cross-Sectional)	Risk/Epi	1426 (33.7%)	Young adults experiencing homelessness	Arizona, California, Colorado, Missouri, New York & Texas	18–26	Co-Occurring	
Omura et al. (2014) [17]	Quantitative (Pros. Cohort)	Risk/Epi	1019 (31.4%)	Street-involved youth who use illicit drugs	Vancouver, Canada	14–26	H → J	
Pilnik et al. (2017) [18]	Argument	Argument	N/A	Unaccompanied homeless youth; Justice-involved youth	N/A	N/A	Co-Occurring	
Quirouette et al. (2016) [19]	Ethnography	Ethnography	51 (51%)	Homeless and street-involved youth	Ontario & Nova Scotia, Canada	17–25	J → H	
Ryan et al. (2007) [20]	Quantitative (Cross-Sectional)	Risk/Epi	294 (0%)	Male adolescents leaving foster care	Midwestern U.S. (unspecified)	16–22	H → J	
Schoenfeld et al. (2019) [21]	Qualitative	Program Evaluation	19 (42.1%)	Homeless youth	Texas	19–26	Co-Occurring	
Shah et al. (2017) [22]	Mixed-Methods	Risk/Epi	1202 (54%)	Youth and young adults exiting child welfare system	Washington	17–21	J → H	
Snyder et al. (2016) [23]	Theoretical	Theoretical	N/A	Homeless youth	N/A	16–24	H → J	General Strain Theory; Traumatic Stress Theory
Tam et al. (2016) [24]	Quantitative (Cross-Sectional)	Risk/Epi	272 zip codes (% N/A)	Transition age youth exiting public systems	California	18–25	Co-Occurring	
Thrane et al. (2008) [25]	Quantitative (Cross-Sectional)	Risk/Epi	361 (% unknown)	Homeless youth in the U.S. Midwest	Iowa, Kansas, Missouri, Nebraska	16–19	H → J	Developmental Theory; Social Interaction Theory
Vidal et al. (2017) [26]	Quantitative (Prospective Cohort / Longitudinal)	Risk/Epi	10,850 (46%)	Maltreated children and adolescents referred to child protective services	Rhode Island	2–13	H → J	Cycle of Violence Theory; Cumulative Risk Theory
Walker et al. (2018) [27]	Mixed Methods	Observational	13,657 (% unknown)	Court-involved youth	Washington	12–17	Co-Occurring	
Wendy & Rossman (2011) [28]	Argument	Argument	N/A	Children in juvenile delinquency cases	USA (nationwide)	N/A	J → H	
Yoder et al. (2014) [29]	Quantitative (Cross-Sectional)	Risk/Epi	202 (36.1%)	Homeless youth with childhood trauma	Midsized western city (U.S.)	18–24	H → J	

### Characteristics of included studies

Publications included in this review were predominantly quantitative analyses (18/29; See Table 2, articles 1, 2, 5, 6–8, 10–14, 16, 17, 20, 24–26, 29), with the vast majority of these featuring a cross-sectional study design (12/18; 1, 2, 5, 6, 8, 12, 14, 16, 20, 24, 25, 29). The remaining quantitative studies included three longitudinal studies (10, 11, 13), two prospective cohort studies (17, 26), and one program evaluation (7). The majority of publications (17/29; 1, 2, 5, 6, 8, 10–13, 16, 17, 20, 22, 24–26, 29) took a risk-based epidemiological approach to either predicting housing instability or justice system contact. Three publications were ethnographic studies of street-involved youth (3, 15, 19), with an additional three publications classified as non-empirical “arguments” advocating for a particular set of policies (4, 18, 28). Two studies incorporated mixed methods in their analyses (22, 27), while one qualitative program evaluation was identified (21). We identified only one systematic review (9) and one publication was classified as theoretical in nature (23). To assess the prevalence of the direction of estimated effects, we coded the studies according to the direction of causality implied or stated by the papers. These effects include 1) housing instability as a predictor of justice system contact, 2) justice system involvement as a predictor of housing instability, or 3) co-occurring housing instability and justice system contact. (Table 3).

**Table 3:** Characteristics of Included Studies

Publication	Target Sample	Findings
*3.1 - Housing instability as a predictor of justice system contact*		
Baron (2008)	Actively homeless or runaway youth	Anger over unemployment attributed to unjust external forces predicts violent crime and drug dealing among homeless youth. Such anger is conditional upon subjective interpretation of a youth’s economic deprivation.
Baron (2016)	Actively homeless or runaway youth	Low self-control among homeless youth is associated with stronger deviant attitudes, greater association with delinquent peers, likelihood of legal involvement, and more contact with law enforcement. However, self-control is not associated with duration of homelessness.
Boyd et al. (2016).	Actively homeless or runaway youth; Identity- or behavior-specific populations	Accumulation of adverse events over the life course may better predict negative health and social outcomes than any one incident. Canadian state interventions (e.g., child apprehension, foster care, enactment and enforcement of harsh drug and anti-homelessness laws, etc.) reinforce and reproduce the structural violence of racialized and socioeconomic inequalities, which particularly harm First Nations homeless youth.
Chapple et al. (2004)	Actively homeless or runaway youth; Survivors of abuse, trauma, or mental disorder	Factors associated with self-reported offending (e.g., sexual abuse, having deviant peers, etc.) are similar to factors associated with arrest among homeless and runaway youth. Associations are greater for boys than girls despite girls reporting higher levels of household trauma prior to runaway. Duration of homelessness is not associated with arrest.
Chen et al. (2006)	Actively homeless or runaway youth; Survivors of abuse, trauma, or mental disorder	Involvement with the criminal legal system is associated with certain lifetime mental disorders. Externalizing disorders (e.g., substance abuse and conduct disorder) are related to arrest. Street youth with comorbid externalizing and internalizing disorders (e.g., depression, posttraumatic stress disorder) are more likely to be arrested than nondisordered youths. No significant association exists between youth with only internalizing disorders and nondisordered youths.
Courtney et al. (2019)	Youth exiting systems	Pre-transition support for youth exiting the child welfare or juvenile justice systems is positively associated with housing stability, employment, income, health, and safety, but has no impact on measures of education, social support, delinquent behavior, or justice system involvement.
Crawford et al. (2018)	Youth exiting systems; Survivors of abuse, trauma, or mental disorder	Males exiting the foster care system with mental health service needs are most at-risk for serious criminal involvement as an adult and may benefit from early prevention and intervention services before the transition to adulthood. Increased placement instability in the form of number of placements and runaways were related to higher odds of an adjudicated felony.
Edalati & Nicholls (2019)	Actively homeless or runaway youth; Survivors of abuse, trauma, or mental disorder	Exposure to childhood maltreatment (e.g., abuse, neglect) is one of the most significant predictors of justice involvement and victimization among homeless populations. Physical and sexual abuse are particularly associated with increased risk of justice involvement and victimization regardless of demographic, mental health, or delinquent behavior (e.g., substance use).
Ivanich & Warner (2019)	Actively homeless or runaway youth; Identity- or behavior-specific populations	Non-White youth facing housing instability are more likely than White youth to report being harassed by the police. However, White homeless youth living directly on the street (or in abandoned buildings) are just as likely to experience police harassment as non-White homeless youth, indicating homelessness may be a “master status” superseding the effect of race on police contact.
Jackson et al. (2017)	Housed but risk for housing instability	Adverse housing conditions (i.e., disarray, deterioration, and health/safety hazards) are associated with significant increases in early-onset delinquency and significant increases in the odds of severe early-onset delinquency. Severe early-onset delinquency among children exposed to housing risks in the presence of health/safety hazards is nearly four times larger than such delinquency among unexposed children.
Jeanis et al. (2019)	Actively homeless or runaway youth; Survivors of abuse, trauma, or mental disorder	Runaways are a heterogeneous group with highly unique experiences and risk factors occurring before and after the runaway experience. Classifying runaway type based solely upon motivation, individual characteristics, victimization, or offending is too narrow a perspective. ‘Impulsivity’ represents a novel typology in the classification of runaway youth. Incorporating sub-types into analyses may better identify typologies most likely to offend.
Kolivoski et al. (2017)	System-involved youth	Out-of-home placement in child welfare system is associated with legal system involvement. Youth with chronic justice system involvement have more experiences in group homes and residential facilities. Those with less frequent justice system contact tend to have foster home experiences.
McCandless (2018)	Formerly homeless individuals; Identity- or behavior-specific populations	LGBTQ+ homeless youth represent a vulnerable population reporting both fear of and harassment by law enforcement, including fear of being sent back to an abusive household. These youth also report barriers to accessing services, including shelters.
Omura et al. (2014)	Actively homeless or runaway youth; Identity- or behavior-specific populations	Youth who are homeless, substance users, or engaged in risky behaviors (e.g. public injection and drug dealing) are significantly more likely to be recently incarcerated.
Ryan et al. (2007)	Youth exiting systems; Identity- or behavior-specific populations	Adolescents leaving foster care face elevated risk of offending if not enrolled in school. Placement instability, placement upon exit, and prior arrest are associated with increased risk of delinquency.
Snyder et al. (2016)	Actively homeless or runaway youth; Survivors of abuse, trauma, or mental disorder	Polyvictimization, multiple system involvement, and LGBTQ+ identity represent strains especially relevant to homeless youth, which may help explain their high risk of justice system involvement.
Thrane et al. (2008)	Actively homeless or runaway youth; Survivors of abuse, trauma, or mental disorder	Substance use and having delinquent peers are associated with police harassment but not arrest, whereas first runaway occurrence is associated with arrest. Physically abused youth encounter more police harassment, while minor delinquent behavior increases risk of arrest.
Vidal et al. (2017)	Youth exiting systems; Survivors of abuse, trauma, or mental disorder; Identity- or behavior-specific populations	Social risk factors (i.e., age, gender, race/ethnicity), recurrence of maltreatment, experiencing at least one incident of neglect, and family poverty significantly predict risk of juvenile justice system involvement. However, subtypes of maltreatment, including physical, sexual, and other types of abuse do not significantly predict the risk of juvenile justice system transition.
Yoder et al. (2014)	Actively homeless or runaway youth; Survivors of abuse, trauma, or mental disorder	Exposure to childhood physical abuse predicts arrest and jail admission even after accounting for a homeless youth’s level of substance use, interactions with deviant peer groups, and engagement in survival behaviors on the streets. Initial involvement in the justice system is associated with youths’ attempts to make money and find resources to survive on the streets. High-risk survival behaviors, while predictive of arrest, are less important in predicting involvement in more severe levels of the criminal legal system.
*3.2 - Justice system involvement as a predictor of housing instability*		
Britton & Pilnik (2018)	Youth exiting systems	Pre-release interventions reduce the likelihood of homelessness upon system exit. Courts have the ability to prevent, alleviate, and/or end homelessness for youth who appear before them via strategies presented in National Council of Juvenile and Family Court Judges resolutions.
Quirouette et al. (2016)	Actively homeless or runaway youth	Exposure to the legal system (e.g., via arrest, court/jail records, mandated oversight) creates short- and long-term systemic barriers to unhoused youth obtaining stable housing, education, and/or employment. Such exposure also shapes a youth’s individual self-perception, motivation, and hope for the future. These combined effects negatively impact the ability of youth to transition away from homelessness, lengthen the process for securing stability, and threatening the youth’s overall well-being and ability to access opportunities for upward mobility and autonomy.
Shah et al. (2017)	Youth exiting systems	Youth who experience disrupted adoptions, have multiple foster care placements (especially in congregate care settings), or are involved with the juvenile justice system are more likely to become homeless. Court- involved youth who have four or more convictions or adjudications in the last 24-month period are more likely to experience homelessness.
Wendy & Rossman (2011)	Actively homeless or runaway youth; System-involved youth	Punitive housing policies in response to juvenile delinquency strain parent-child relationships and increase the likelihood of homelessness for children and families. Decisions about the status of the child in juvenile court should be made with full knowledge of how those decisions impact the possibility of eviction and future housing instability for the youth and their family.
*3.3 Co-occurring housing instability and justice system contact*		
Kort-Butler & Tyler (2011)	Actively homeless/runaway youth	Nearly two-thirds of youth in the incarceration cluster had been kicked out by a parent/caretaker, compared to less than half the youth in the other three clusters. A portion of street youth were more likely to have their behavior criminalized instead of being able to access available legitimate street resources. Youth may be shut out of legitimate resources as a result of incarceration histories.
Narendorf et al. (2020)	Actively homeless or runaway youth; System-involved youth	Homeless youth exposed to the juvenile justice system, either alone or in combination with the foster care system, had high rates of childhood trauma exposure and also presented increased risk for substance use and arrest in young adulthood. Those with dual status involvement were at highest risk for engaging in survival sex and experiencing an unplanned pregnancy, in addition to substance use and arrest.
Pilnik et al. (2017)	Actively homeless or runaway youth; System-involved youth	Youth involvement with the justice system can increase the likelihood of future homelessness for many reasons, including the fact that educational disruptions and juvenile delinquency records can make it harder to obtain employment. Youth experiencing homelessness may also be swept into the juvenile justice system through laws that prohibit simply being in public spaces, such as juvenile curfews, or anti-sitting or sleeping ordinances.
Schoenfeld et al. (2019)	Actively homeless or runaway youth; System-involved youth	Homeless youth in Austin, Tx, are historically excluded from. More than 75% of homeless youth in Austin, TX, have a history of involvement with foster care or the juvenile justice system. However, these youth have historically been excluded from providing input into system-planning efforts directed toward engaging homeless youth. Treating youth as equal partners gives them a seat at the table in a system that has largely failed them, and empowers youth to influence community-wide decision-making that affects homeless youth populations.
Tam et al. (2016)	Youth exiting systems	Transition age youth (TAY) exiting the child welfare and juvenile justice systems in Los Angeles, CA, experience high rates of homelessness. Locations of beds in shelters and/or housing facilities are not related to the zip codes where youth are transitioning out of foster care or the juvenile justice systems. Further, regardless of whether they are TAY-specific, all beds exist in low-income zip codes that do not support TAY’s transition to adulthood.
Walker et al. (2018)	Actively homeless or runaway youth	Families are frustrated with the perceived inadequacy of available justice responses to home conflict and youth intractability, as services are available for youth and families with lower needs but not complex issues. Court processes may create additional barriers, such as issuing postal mail warrants to youth without a fixed address. Courts are ill-equipped to identify housing unstable youth via existing intake assessments, and should actively incorporate pre-transition planning for youth exiting the justice system into community services and re-entering public systems (e.g., the school system).

### Housing instability as a predictor of justice system contact

We identified 19 articles in which housing instability was demonstrated to increase the likelihood of youth contact with the justice system. Contact was defined as arrest, re-arrest, or police contact. The housing status of youth in these articles varied, with two-thirds of studies focused actively homeless youth (1–3, 5, 6, 9, 10, 11, 12, 15, 17, 23, 25, 29), whereas four publications highlighted youth transitioning from out-of-home placements, (e.g., custodial care, child protective services) into unstable housing situations (7, 8, 20, 26). One study assessed the effects of out-of-home placement types on justice contact as an early indicator of housing instability that resulted in later criminal-legal involvement (13).

The four articles examining the risk of housing instability for youth exiting out-of-home placement systems included transitions from foster care (8, 20) and child protective services (7, 26). These studies demonstrated that youth with unstable placement histories (e.g., multiple foster home or custodial care placements) were more likely to encounter the criminal legal system in adulthood. Meanwhile, pre-transition planning support was associated with later stable housing, employment, and self-reported health.

The studies in this category tended to view housing status as one of multiple risks for later criminal legal involvement. For example, eight of the 19 studies in this category identified exposure to abuse and/or trauma in youth or childhood as a risk related to housing instability and elevated justice system contact (5, 8, 9, 12, 23, 25, 26, 29). Six articles highlighted youth-identity characteristics, such as ethnicity and sexual or gender minority status, as factors associated with increased justice system contact among actively homeless youth (3, 10, 15, 23, 25, 26). The presence of mental health disorders (6, 8, 12) or use of illicit substances (3, 17, 20) were also identified as factors associated with an elevated risk of justice system contact either as a correlate or predictor of housing instability.

### Justice system involvement as a predictor of housing instability

Four of the 29 articles identified contact with the justice system as a predictor of homelessness or housing instability. Two publications highlighted the housing challenges facing youth exiting the juvenile justice system (4, 22), and two articles focused on the cycling of actively homeless youth in and out of the juvenile justice system (19, 28). In all studies, the authors argued that legal system involvement presents active structural barriers to youth obtaining housing. Structural barriers include difficulties securing housing leases for youth with criminal records and the acquisition of a criminal record triggering risks of eviction from stable housing. Additionally, involvement with the legal system adversely impacts a youth’s self-perceptions and motivation toward self-improvement, resulting in lengthier timelines for securing stable housing and decreased reports of overall health and well-being (19). Transition planning prior to exit from the justice system was associated with a greater likelihood of youth remaining housed following exit (4, 22).

### Co-occurring housing instability and justice system contact

Six articles focused on the reciprocal associations between housing instability and justice system contact. Five articles focused on actively homeless youth (14, 16, 18, 21, 27). Findings from these studies indicate that homeless youth, particularly those engaged in street survival behaviors, were at elevated risk of being subject to punitive law enforcement actions, rather than being linked resources or services in their communities. Involvement with law enforcement or the juvenile justice system further increased the likelihood of these youth becoming homeless. Two articles (16, 24) focused on youth exiting systems. Findings from these studies indicated that youth involved with multiple systems, such as out-of-home placement and the justice system, faced higher odds of housing instability upon exit from these systems. Involvement in one of these systems elevated the likelihood that youth will be involved in the other system as well. However, youth who were actively linked to post-system housing options and relevant social services were more likely to obtain stable housing and avoid future contact with the justice system. Three studies employed quantitative methods (14, 16, 24), two papers incorporated qualitative methods (18, 21), while one article provided a mixed-methods analysis (27).

### Theoretical frameworks informing research

Of the 29 studies reviewed, only seven clearly identified a theoretical framework(s). The theories referenced fall into two broad categories, with five articles referencing a criminological theory (1, 2, 10, 11, 23), one referencing developmental theories (25), and one (26) engaging both criminological and developmental theories. Notably, none of the 29 articles explicitly referenced explicit social determinants theories or frameworks.

Among criminological theories, General strain theory (GST) was the only theory referenced by multiple publications (1, 11, 23). Each of these publications applied GST to interpret the experiences of youth whose perceptions of injustice and unfairness led the youth into the commission of delinquent activity. For example, Baron (2008) used GST to extend the research on unemployment and crime by articulating how 1) unemployment increases a homeless youth’s anger and the perception that their circumstances derive from injustice or unfairness in the market, and 2) when sustained over time, this anger serves to contract the homeless youth’s social interactions to peers in similar circumstances, elevating the likelihood that the youth may acquire deviant peers and increase their risk of justice system contact. Meanwhile, Jackson et al. (2017) note that when criminological theories have engaged issues of housing, including GST, they focus on macro-level factors such as neighborhood or social disorder, rather than micro-level factors such as the ecology of the proximal housing conditions in which a child grows up. The authors note that these dynamics must be considered using a GST lens when looking at youth delinquency.

Finally, Snyder et al. (2016) use the overarching framework of GST to identify multiple key strains that may contribute to delinquent behavior among homeless youth. These include 1) polyvictimization (i.e., exposure to multiple compounding forms of violence, crime, or abuse) that results in trauma, anger, and other emotions that may contribute to offending behavior; 2) discrimination and violence as a result of identifying as a sexual or gender minority (i.e., LGBTQ+ identity), including being kicked out of one’s home and/or being physically or sexually abused while homeless; and 3) multiple system involvement (i.e., child welfare, juvenile justice, mental health, and substance abuse treatment) and the inability of service agencies to meet the complex needs of youth exiting systems. The authors note that while GST focuses on strain and hardship, it is also a framework that highlights resilience to adversity and delinquency. Resilience for homeless youth may develop from access to services that meet immediate needs (e.g., food, shelter, etc.), and provide coping resources and positive social support (e.g., mentorship, positive adult interactions, support for a youth in achieving particular goals).

Vidal et al. (2017) applied the cycle of violence theory to illustrate how exposure to adverse experiences elevates the risk of justice contact among adolescents, with engagement in crime and delinquency identified as learned behaviors stemming from exposure to abuse rather than endogenously emerging from the delinquent youth. Meanwhile, Baron (2016) challenged existing self-control theory, contending that the deviant peers of homeless youth may account for youth experiences with law enforcement more than youth having low impulse control. Additional theories, such as focal concerns theory and broken windows theory that seek to explain decision-making motivations and tactics by law enforcement, were referenced by Ivanich & Warner (2019). However, the authors did not substantively incorporate these theories into their hypotheses or analysis beyond references included in a review of the existing literature.

Among the developmental theories referenced, Thrane et al., (2008) referenced the risk amplification model (Hoyt & Whitbeck, 1999), which is a fusion of developmental theory and social interaction theory (Patterson, 1982). This model holds that youth raised by abusive or criminally involved caregivers 1) are disproportionately set on a trajectory toward running away from such environments, 2) exchange their parental/caregiver relationships for deviant peer networks, and 3) that the combination of the two elevate a youth’s reliance on survival strategies on the street and engagement in deviant behavior, which elevate the risk of justice system contact. Separately, Vidal et al. (2017) frame their work through the lens of cumulative risk theory, positing that the accumulation of adverse childhood experiences, such as exposure to abuse, household violence, parental substance use, poverty, etc., compound the likelihood that a child may be removed from their home and/or experience multiple transitions in and out of the child welfare system. These removals and transitions subsequently expose the child to additional risk factors during their critical developmental years, further compounding the likelihood that the child will be involved in delinquent behavior in their adolescence.

## Discussion

This scoping review sought to examine the integration of social determinants of health theories within the juvenile delinquency research literature. Our study found that a social determinants lens is not well integrated in the applied criminology research literature. The majority of studies identified in our review were focused on the legal system contact of homeless youth and only a handful of articles (four) sought to understand youth delinquent behavior within a context of housing stability. Epidemiology and risk-specific research, rather than research focused on services, was much more likely to explicitly address housing and other social determinants, such as poverty, in the context of youth legal involvement. The lack of continuity in theorizing from epidemiology to applied science in this area is a gap that is likely reducing the effectiveness of interventions to interrupt patterns of legal system contact for youth. We discuss the implications of these findings below.

In our review, housing figured most prominently in epidemiological studies predicting risk but relatively less in intervention studies of delinquency-crime reduction. We found very few intervention studies focused on recidivism reduction that centered housing as a key component of the service model. When engaged, housing was considered as a component of broader reentry services for youth who had been detained away from home (McCandless, 2018) or had aged out of an out-of-home placement situation (Britton & Pilnik, 2018; Courtney et al., 2019; Crawford et al., 2018; Kolivoski et al., 2017; McCandless, 2018; Shah et al., 2017; Vidal et al., 2017), rather than as a risk to be addressed at the front-end of justice contact.

Unexpectedly, few studies identified specific theories guiding the research, while no publications made explicit reference to public health theories more broadly or social determinants of health frameworks in particular. When referenced at all, theories were more likely to derive from criminology frameworks (e.g., General Strain Theory). Studies incorporating developmental theories (Thrane et al., 2008; Vidal et al., 2017) largely focused on the ways in which poverty and other stressors amplify risks for delinquency, with homelessness being a risk amplifier (e.g., exposure to defiant peers, risk of school disengagement, risk of abuse or trauma, etc.), rather than a determinant of various behaviors or justice contact in and of themselves. Apart from two studies of youth reentry after discharge from physical detention, we found no empirical studies of the effects of legal system actions on the housing status of youth. Policy and position papers articulated a number of potential areas for legal system reform to mitigate the impact of legal involvement on youth housing, however, we found no studies that tested the impact of current or alternative processes on youth housing outcomes (e.g., legal system involvement as a direct risk for becoming unhoused).

The lack of applied studies in this review of housing and legal system involvement reflects the cognitive-centered orientation predominant in delinquency intervention and prevention literature. The dominant framework guiding criminology service research is the risk-needs-responsivity (RNR) model (Andrews, Bonta & Hoge, 1990a; Andrews, Zinger, et al., 1990). This model was developed to respond to the prevailing, retributive-focused policies of the late twentieth century. During the 1980’s and 1990’s, policymakers responded to public concern about rising crime rates by enacting harsher dispositions and sentencing laws that implicitly or explicitly rejected the idea of a rehabilitative criminal-legal system (Metze, 2014). The result was mass incarceration and the RNR framework was intended as a progressive effort to divert adult offenders from prison sentences (Andrews, Bonta & Hoge, 1990a; Andrews, Zinger, et al., 1990) by providing community-based services that would be (1) more cost-effective (Taxman & Marlowe, 2006) and (2) rehabilitative rather than punitive in nature. The separation of risk and need in the model reflects a distinction between specific factors that predispose a person to delinquent behavior (risk) and the ecological factors that are needed to function in society (need).

Social determinants frameworks, on the other hand, conceptualize individual behaviors within the wider context of ecological determinants, or the “needs” part of the RNR model. Rather than focusing on proximal risks or behaviors, these frameworks look upstream at distal factors that shape individual behaviors (Bronfenbrenner, 1977). As we see from the epidemiological literature, the immediate infractions of survival behaviors of homeless youth serve as proximal catalysts for arrest and subsequent involvement in the legal system. These behaviors are rooted in ecological contexts that significantly shape that youth’s immediate needs (e.g., food, shelter, money), behaviors (e.g., survival sex, trespassing, petty theft), and resulting outcomes (e.g., contact with police, arrest, obtaining a criminal record). Expanding the “risk” component of the RNR framework to meaningfully encompass social determinants such as housing is likely to have transformative effects on the scope of services that can be funded through existing allocation to justice-related budgets. Social ecological theory is a strong influencing factor in theories of youth risk and protective behaviors (Crosby et al., 2018; Nooe & Patterson, 2010), and our review highlights the limited influence this theory has had in applied criminology. This is likely due in part to the expense of providing housing support, the view that housing would be outside of the purview of the justice system’s responsibility, and the inconsistent influence of social welfare and developmental theory on adolescent criminological theory.

Public health scholarship has much to offer the criminological literature. First, applying ecological frameworks (Bronfenbrenner, 1977) to youth behavior, with the acute recognition of adolescence as a “sensitive/critical period,” (Keyes & Galea, 2016; Kuh et al., 2003; Wood et al., 2018) may accelerate the re-framing of significant aspects of youth delinquent behavior not as unique and isolated delinquent activities, but rather as manifestations of links in a chain of risk (Ben-Shlomo & Kuh, 2002; Keyes & Galea, 2016). Rather than criminalizing various “survival” behaviors emanating from housing instability (Yoder et al., 2014), the application of a social determinants lens would identify housing instability as an upstream determinant of such behaviors, with a resulting demand for the direction of resources to engage the root of the problem (i.e., housing instability) rather than the symptoms manifesting as delinquent behavior.

Future research should incorporate multiple approaches to engaging the intersection of youth housing instability and contact with the criminal legal system. First, studies are needed to investigate the degree to which strengthening social determinants of health alone are sufficient for reducing (re)offending. As survival behaviors draw the attention of law enforcement and elevate risk of legal system contact, engaging the upstream determinants (e.g., secure housing) that minimize the need for such behaviors is critical. Second, a need for more intervention-focused studies exists. Few studies in this review engaged the role of community-based resources or programs in supporting housing unstable youth and/or youth exiting the legal system. Shifting the current research focus from risk-focused epidemiological studies to assessments of practical interventions will help identify tangible mechanisms by which to strengthen the determinants of health for youth at risk of housing instability or justice contact. Finally, studies are needed to articulate the breadth of experiences of justice involved youth and the ways in which upstream determinants uniquely predict justice involvement over and above the current presumed mechanisms in the criminological literature, such as having antisocial peer networks. Such a research agenda will shift the focus from the individual and their specific behaviors toward the wider ecology of risk and protective factors in which those behaviors occur.

### Limitations

Our review is limited by two key factors. First, consistent with the standards for scoping reviews, we restricted our search terms to the title, abstract, or keyword of publications. Thus, we may have missed articles highlighting interventions that address housing as a peripheral service. Second, we intentionally limited our review to the subject of housing. As such, we may have missed important studies regarding other critical and intersecting social determinants affecting justice-involved youth, such as economic stability, employment, social capital, among others engaged in the literature.

## Conclusion

This review found that a social determinants lens is not well integrated in the applied criminology research literature. Publications are primarily focused on the social epidemiology of risk factors and behaviors determining youth justice contact, but relatively less so on studies of interventions targeting youth delinquency, crime reduction, or recidivism with regard to housing as an upstream determinant of these outcomes. A lack of continuity in theorizing from epidemiology to applied science in this area represents a gap in the literature, and, potentially, by extension in practice, that is likely reducing the effectiveness of interventions to interrupt patterns of legal system contact for youth. Integrating a public health framework that emphasizes the upstream social determinants leading to contact with the youth justice system would represent a paradigm shift for the field that would have beneficial effects on long term health outcomes for youth.

## Data Availability

Not applicable.
